# Integrative analysis of dysregulated lncRNA-associated ceRNA network reveals potential lncRNA biomarkers for human hepatocellular carcinoma

**DOI:** 10.7717/peerj.8758

**Published:** 2020-03-11

**Authors:** Chengyun Li, Wenwen Zhang, Hanteng Yang, Jilian Xiang, Xinghua Wang, Junling Wang

**Affiliations:** 1 Department of Toxicology, School of Public Health, Lanzhou University, Lanzhou, Gansu province, China; 2 Department of General Surgery, Lanzhou University Second Hospital, Lanzhou, Gansu province, China; 3 Department of gastroenterology, Third People’s Hospital of Gansu province, Lanzhou, Gansu province, China; 4 Department of gastrointestinal surgery, Gansu Wuwei Tumor Hospital, Wuwei, Gansu province, China

**Keywords:** Hepatocellular carcinoma, LncRNA, ceRNA, Clinical features, Overall survival

## Abstract

**Background:**

Hepatocellular carcinoma (HCC) is an aggressive cancer with a poor prognosis and a high incidence. The molecular changes and novel biomarkers of HCC need to be identified to improve the diagnosis and prognosis of this disease. We investigated the current research concentrations of HCC and identified the transcriptomics-related biomarkers of HCC from The Cancer Genome Atlas (TGCA) database.

**Methods:**

We investigated the current research concentrations of HCC using literature metrology analysis for studies conducted from 2008 to 2018. We identified long noncoding RNAs (lncRNAs) that correlated with the clinical features and survival prognoses of HCC from The Cancer Genome Atlas (TGCA) database. Differentially expressed genes (lncRNAs, miRNAs, and mRNAs) were also identified by TCGA datasets in HCC tumor tissues. A lncRNA competitive endogenous RNA (ceRNA) network was constructed from lncRNAs based on intersected lncRNAs. Survival times and the association between the expression levels of the key lncRNAs of the ceRNA network and the clinicopathological characteristics of HCC patients were analyzed using TCGA. Real-time polymerase chain reaction (qRT-PCR) was used to validate the reliability of the results in tissue samples from 20 newly-diagnosed HCC patients.

**Results:**

Analysis of the literature pertaining to HCC research revealed that current research is focused on lncRNA functions in tumorigenesis and tumor development. A total of 128 HCC dysregulated lncRNAs were identified; 66 were included in the co-expressed ceRNA network. We analyzed survival times and the associations between the expression of 66 key lncRNAs and the clinicopathological features of the HCC patients identified from TCGA. Twenty-six lncRNAs were associated with clinical features of HCC (*P* < 0.05) and six key lncRNAs were associated with survival time (log-rank test *P* < 0.05). Six key lncRNAs were selected for the validation of their expression levels in 20 patients with newly diagnosed HCC using qRT-PCR. Consistent fold changes in the trends of up and down regulation between qRT-PCR validation and TCGA proved the reliability of our bioinformatics analysis.

**Conclusions:**

We used integrative bioinformatics analysis of the TCGA datasets to improve our understanding of the regulatory mechanisms involved with the functional features of lncRNAs in HCC. The results revealed that lncRNAs are potential diagnostic and prognostic biomarkers of HCC.

## Introduction

Hepatocellular carcinoma (HCC) ranks sixth, worldwide, in cancer incidence and fourth in mortality with 841,000 new cases and 782,000 deaths, annually (Bray et al., 2018). Approximately 75–85% of all liver cancer cases are classified as HCC and 10%–15% of cases are intrahepatic cholangiocarcinoma, according to Global Cancer Statistics 2018 from the International Agency for Research on Cancer. The most common risk factors for HCC are chronic hepatitis B virus (HBV) or hepatitis C virus (HCV) infection, heavy alcohol intake, obesity, type 2 diabetes, and smoking (Lu et al., 2016a; Lu et al., 2016b; Suh et al., 2018). Many studies have revealed that the occurrence and development of HCC are associated with abnormal genetic changes and cancer-related signaling pathways (Koh et al., 2018; Peng et al., 2019; Pinato et al., 2017). Most HCC patients are diagnosed when the disease is already at an advanced stage or has progressed to lymphatic metastasis (Shiani et al., 2017). The prognosis for advanced HCC is poor and an insufficient number of biomarkers have been identified for the early diagnosis and prognosis of this disease (Yasuda et al., 2019). The identification of more accurate HCC diagnostic and prognostic biomarkers is needed to improve the early diagnosis of HCC and the prognostic classification of this disease.

Recent advancements in high-throughput gene sequencing analysis have led to the identification of a large number of differentially expressed long non-coding RNAs (lncRNAs) in the progression of various cancers (Jing et al., 2018; Li et al., 2018; Malih, Saidijam & Malih, 2016). A growing number of studies have reported that dysregulated lncRNAs in HCC are related to chronic HBV or HCV infection, histological type, TNM stage, lymph node metastasis, and prognosis (Hu et al., 2018; Motawi et al., 2019; Zheng et al., 2017). Closely related dysregulated lncRNAs may assist in identifying valuable biomarkers for the diagnosis and prognosis of HCC. Current studies of HCC and lncRNAs have mainly focused on sequencing small tissue samples and cell lines (Tan et al., 2019; Zhao et al., 2019a; Zhao et al., 2019b; Zhao et al., 2019c). Large sample population studies are rarely reported. Current research is shifting to focus on the importance of dysregulated lncRNAs in HCC in large sample populations.

High-throughput RNA sequencing technologies are frequently used for the detection of lncRNA alterations in carcinogenesis and in screening for potential biomarkers of numerous diseases. RNA sequencing data was obtained from The Cancer Genome Atlas (TCGA; https://portal.gdc.cancer.gov/) database using microarray chip types and RNA sequencing data standardization quality control (Sanchez-Vega et al., 2018). The identification of lncRNAs that are highly correlated with HCC are reliable when large samples and multiple analyses from different RNA sequencing database portal platforms are used.

The purpose of this study was to investigate current research interests in HCC using literature metrology analysis for studies published from 2008 to 2018. We analyzed the significant differences in RNA expression acquired from TCGA to identify the novel lncRNA signatures for HCC. Genetic functional enrichment analysis was performed based on these lncRNAs, competing endogenous RNA (ceRNA) network construction, differentially expressed lncRNAs, and HCC clinicopathological features correlation and survival analysis. Quantitative reverse transcription polymerase chain reaction (qRT-PCR) was used in the bioinformatics analysis of 20 recently collected HCC tissue samples. This novel approach will improve the discovery of potential lncRNA biomarkers for the diagnosis, classification, and prognostic prediction of HCC.

## Materials & Methods

### Literature metrology analysis method

All HCC-related literature was obtained from the Science Citation Index Expanded (SCI-E) from the Web of Science (WOS) of Clarivate Analytics on February 1, 2019. The documents were analyzed by two independent authors. The literature data retrieval strategy was as follows: title = (‘hepatocellular carcinoma’) or title = (‘hepatocellular cancer’) or title = (‘liver carcinoma’) or title = (‘liver cancer’) and title = (‘human’) and title = (‘biomarker’). All references were dated between 2009 to 2018 and only research articles and reviews in English were included. The data were obtained from the WOS and did not include animal studies.

HCC biomarker-related literature were collected from the WOS and analyzed using VOSviewer 1.6.5 software (Leiden University, Leiden, Netherlands) and CiteSpace V software (Drexel University, Philadelphia, PA, USA), respectively. VOSviewer 1.6.5 and CiteSpace V software were used to perform literature cluster analysis and key word hotspot analysis.

### Patients and samples

**Figure 1:**
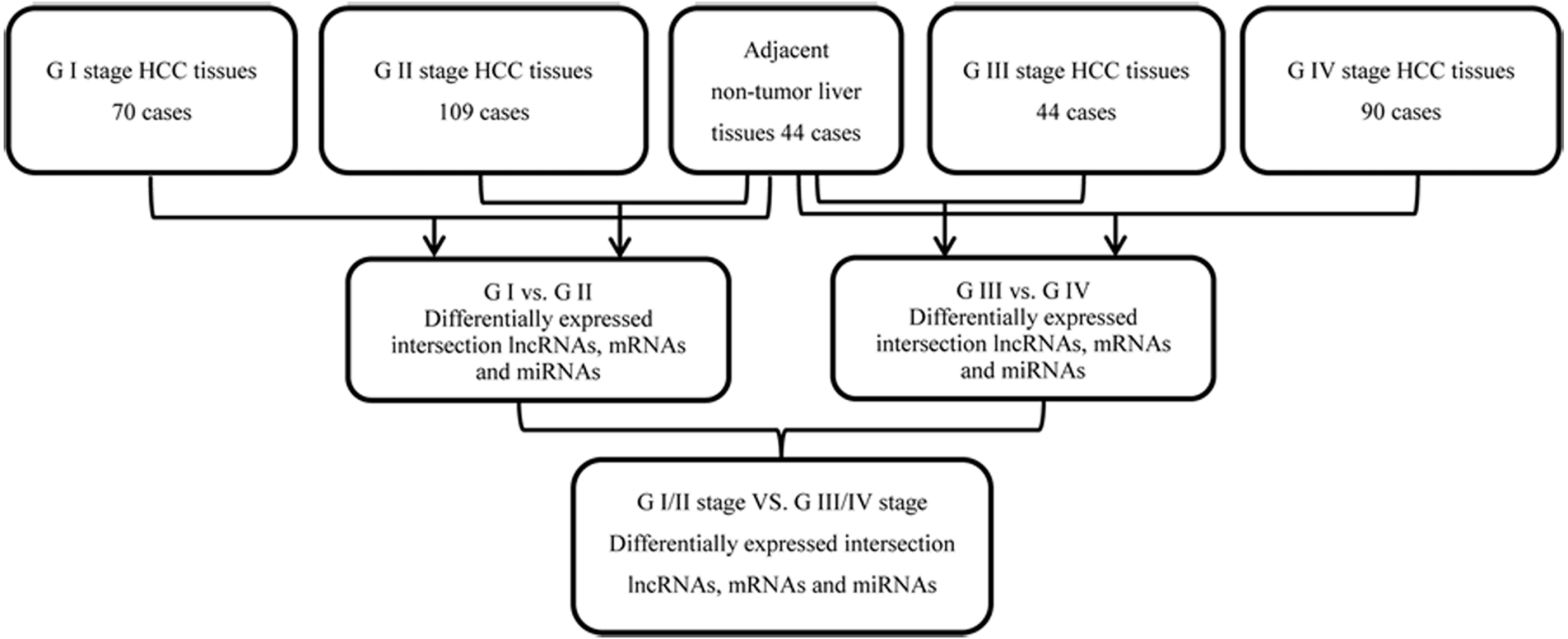
Flowchart for integrated bioinformatics analysis of HCC publicly available RNA sequencing datasets from TCGA database.

We collected data from 349 patients with HCC and RNA sequencing data from TCGA dated up to November 1, 2018. Annotation information for the RNA sequencing datasets were obtained using Affymetrix Human Genome Array platforms. The study was in accordance with the TCGA database portal platform guidelines. The subjects were simplified based on the following exclusion criteria: (a) without completed data information; (b) histologic diagnosis was not HCC; and (c) two or more types of cancers, including HCC. 313 HCC tumors and 44 normal liver tissue samples were included in this study. Of the 313 HCC patients, 233 patients had histopathological stage I/II HCC, and 80 had stage III/IV HCC, according to the 7th American Joint Committee on Cancer (AJCC) Tumor Node Metastasis (TNM) staging system. 249 of these cases had lymphatic metastasis while 64 did not. Details of the RNA sequencing datasets from TCGA, sample descriptions, and clinicopathological features are provided in Table 1. The flow diagram for integrated bioinformatics analysis from TCGA is shown in Fig. 1.

**Table 1:** The clinical information and samples size for TCGA HCC datasets.

**Variable**	**Total cases = 313 (%)**	**Dead cases = 206 (%)**	**Alive cases = 107 (%)**
Gender			
Male	216 (69.00)	145 (70.39)	71 (66.36)
Female	97 (30.99)	61 (29.61)	36 (33.64)
Race			
White	142 (45.37)	82 (39.81)	60 (56.07)
Asia	154 (49.20)	113 (54.85)	41 (38.32)
Black	17 (5.43)	11 (5.34)	6 (5.61)
Age, years			
≤50	149 (47.60)	101 (49.29)	48 (44.86)
>50	164 (52.40)	105 (50.97)	59 (55.14)
Tumor grade			
G I	70 (22.36)	59 (28.64)	11 (10.28)
G II	109 (34.82)	63 (30.58)	46 (42.99)
G III/IV	134 (42.81)	84 (40.78)	50 (46.73)
TNM stage			
I/II	233 (74.44)	185 (89.81)	48 (44.86)
III/IV	80 (25.56)	21 (10.19)	59 (55.14)
Lymph-node status			
No metastasis	64 (20.45)	51 (24.76)	13 (12.15)
Metastasis	249 (79.55)	155 (75.24)	94 (87.85)
Hepatitis B virus chronic infection	54 (100.00)	17 (31.48)	37 (68.52)
Hepatitis C virus chronic infection	73 (100.00)	29 (39.73)	44 (60.27)

### Integration of RNA sequence data and differential expression analysis

RNA level 3 expression data were processed and standardized based on the mRNA expression data of TCGA. The original RNA sequencing raw reads were processed and normalized using the TCGA RNASeqV2 system to fit the analysis. HCC level 3 normalized miRNAs sequencing data (Illumina HiSeq 2000 microRNA sequencing platforms) (https://www.cancer.gov/about-nci/organization/ccg/research/structural-genomics/tcga/using-tcga/types) were also downloaded from TCGA. We analyzed and contrasted the significant dysregulated lncRNAs, mRNAs, and miRNAs in tumor tissues from 313 HCC patients and 41 normal liver tissues using limma R package tool (false discovery rate (FDR)<0.05, fold change>2, *P* < 0.05). We used overlapping subclass analysis to identify the separate dataset co-differentially expressed genes, including lncRNAs, miRNAs, and mRNAs using Venn 2.1 software (http://bioinfogp.cnb.csic.es/tools/venny/index.html). The lists of integrated HCC tissue dysregulated lncRNAs, miRNAs, and mRNAs were saved for further analysis.

### Gene functional enrichment analysis

Significantly dysregulated intersected mRNAs were selected and imported into the Gene Ontology (GO) tool (http://www.geneontology.org) and Kyoto Encyclopedia of Genes and Genomes (KEGG) (http://www.kegg.jp/) to identify their molecular function and to find potential regulated signal pathways for these genes. Up-regulated and down-regulated mRNAs from the overlapping subclasses were analyzed. Visualization of the GO and KEGG were plotted using R software.

### Construction of the ceRNA network

The lncRNA-miRNA-mRNA ceRNA network was built based on the theory that lncRNA can bind miRNA, acting as so-called miRNA sponges, with miRNA binding to the mRNAs and negatively regulating gene expression. HCC tissues significantly dysregulated lncRNAs, mRNAs, and miRNAs (FDR<0.05, fold change >2, *P* < 0.05) and were selected to build the ceRNA network, in which the fold changes of genes were rooted in the TCGA database, to determine whether these intersection genes were involved in ceRNA regulation. MiRcode (https://omictools.com/mircode-tool), miRanda (http://www.microrna.org/microrna/home.do) and Targetscan (http://www.targetscan.org/) were used to predict the miRNA target lncRNAs and miRNA-mRNAs interactions in the different databases. Our study combined the significant function regulation genes in GO and KEGG, and miRNAs predicted target genes to further assist in the selection of the intersection mRNAs. The subset of intersection miRNAs were selected to negatively regulate lncRNAs and to assist in the selection of the intersection mRNAs used to build the ceRNA network according to the ceRNA regulation theory. We used Cytoscape software 3.0 (National Institute of General Medical Sciences, Bethesda, MD, USA) for this analysis.

### Analysis of the association between ceRNA network key lncRNAs and HCC clinical features from TCGA

Abnormally expressed key lncRNAs may play an important role in HCC progression based on the lncRNAs in the ceRNA network. The key lncRNAs involved in the network were selected as target lncRNAs that may be associated with HCC progression. We explored the potential association between ceRNA network key lncRNAs and the TCGA clinical features of HCC patients, which included race, gender, TNM stage, tumor grade, lymphatic metastasis, and chronic HBV or HCV infection, using multiple linear regression analysis.

### Kaplan–Meier survival curve analysis

Kaplan–Meier survival analysis was performed to investigate whether the expression of ceRNA network key lncRNAs was associated with the overall survival of HCC patients. Kaplan–Meier survival analysis parameters were calculated using the publicly available TCGA HCC patient datasets and Gene Expression Profiling Interactive Analysis (GEPIA) tools (http://gepia.cancer-pku.cn/). The survival distributions of patients with HCC in TCGA, and the key lncRNAs expression level changes were analyzed using Kaplan–Meier, log-rank, and hazard ratio (HR). *P* < 0.05 was the cutoff criterion.

### Preparation for human HCC samples and qRT-PCR validation

Samples from tumor tissue and paired non-tumor liver tissue were collected from 20 HCC patients (aged 40–69 years) at Lanzhou University Second Hospital (Lanzhou, China), for qRT-PCR validation. Patients were diagnosed with HCC according to their histopathology. All patients provided informed consent and their clinical information was collected by an investigator using patient interviews and medical records. The collection of the tumor samples from HCC patients was approved by the School of Public Health, Lanzhou University (Lanzhou, China) (Lzuggwsxy-20190806) and conformed to the Helsinki Declaration and current legislation. Samples were collected and stored in RNAlater (Ambion, Foster City, CA, USA) at −80 C.

The total RNA from the tissue samples was isolated using the TRIzol® reagent (Invitrogen; Thermo Fisher Scientific, Inc. Carlsbad, CA, USA). The Reverse Transcription Kit (Promega Corporation, Madison, WI, USA) and GoTaq® qPCR Master Mix of Power SYBR® Green (Promega Corporation) were used to synthesize cDNA and for qRT-PCR detection. qRT-PCR was performed using the Step One PlusTM PCR System (Applied Biosystems; Thermo Fisher Scientific). qRT-PCR relative fold change results were calculated using the 2^−ΔΔCt^ method.

### Statistical analyses

Data were analyzed using SPSS Statistics V21.0 (IBM, Armonk, NY, USA) and expressed as mean ± SD. All analyses were performed three times and represent data from three individual experiments. A two-tailed Student’s *t*-test was used to measure the significance of differences between subgroups. Kaplan–Meier survival analysis was used to investigate the correlation between the changes in lncRNAs expression levels and the prognostic overall survival times for patients. Statistical significance was *P* < 0.05.

## Results

### Literature metrology analysis of HCC research

**Figure 2:**
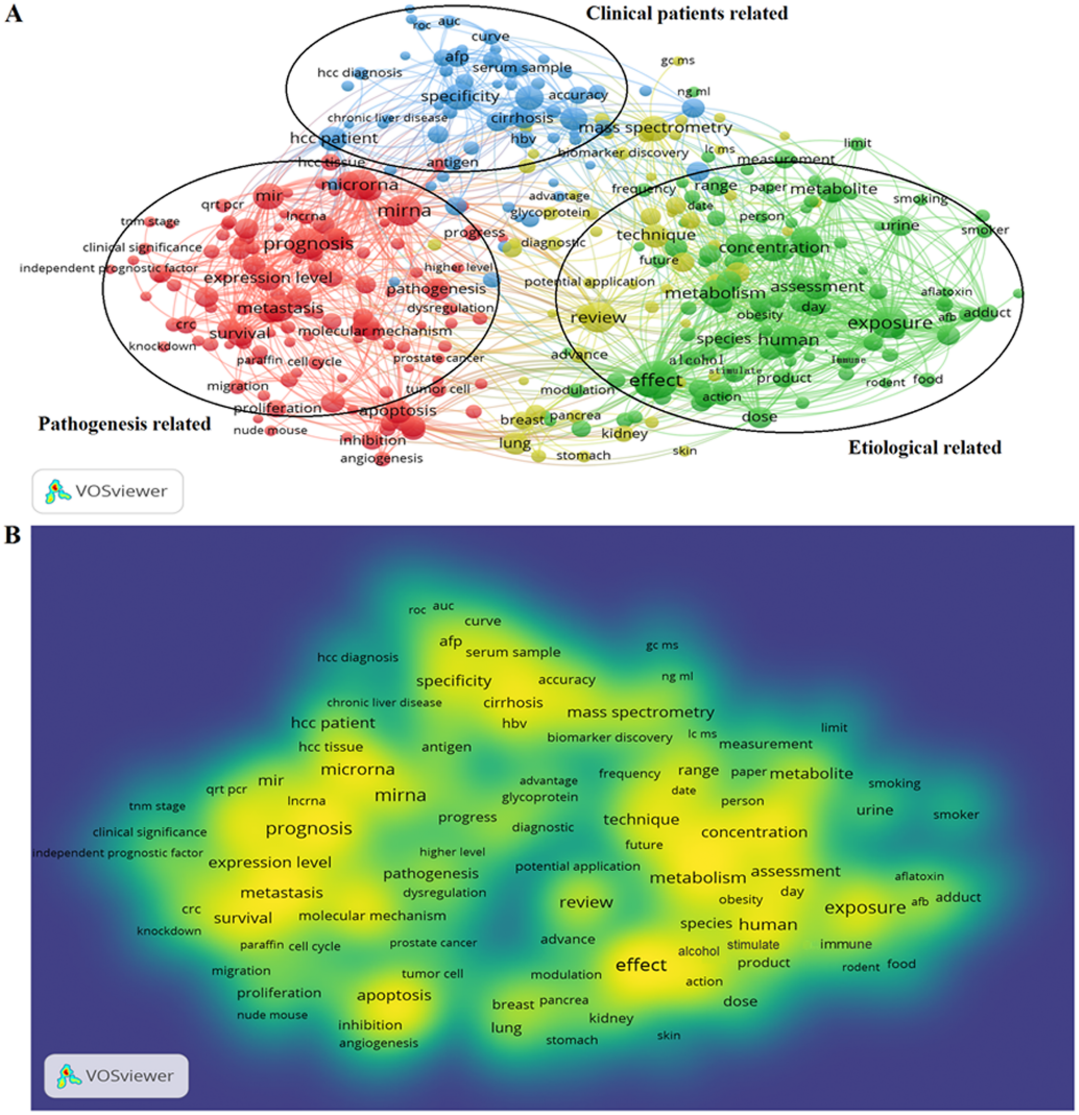
Cluster analysis and hotspot analysis on Hepatocellular carcinoma research. (A) The divided into three clusters: ‘Clinical patients’, related study’, ‘Pathogenesis related study’ and ‘Etiological related study’. The cluster analysis demonstrated that the dominant fields of hepatocellular carcinoma include three research directions. (B) Keywords with high frequency were captured and considered as the hotspots in this field.

922 publications from 2008 to 2018 matched the search criteria. These HCC-biomarker-related studies were analyzed by VOSviewer and three primary clusters were identified: pathogenesis related, clinical patients related, and etiologically related. Cluster analysis showed that there were three major focuses in HCC research (Fig. 2A).

Key words and article titles from the 922 papers were analyzed using VOSviewer software. The integrated analysis is shown in Fig. 2B; colors were assigned to the key words by VOSviewer. The different color shades represent the usage frequency of the key words; the colors, ranging from blue to yellow, represent a low to high frequency of occurrence, respectively. Key words with yellow (high frequency) represented the research hotspots in this field. The key words analysis revealed that “prognosis”, “effect”, “expression”, and “concentration” were frequently used.

**Figure 3:**
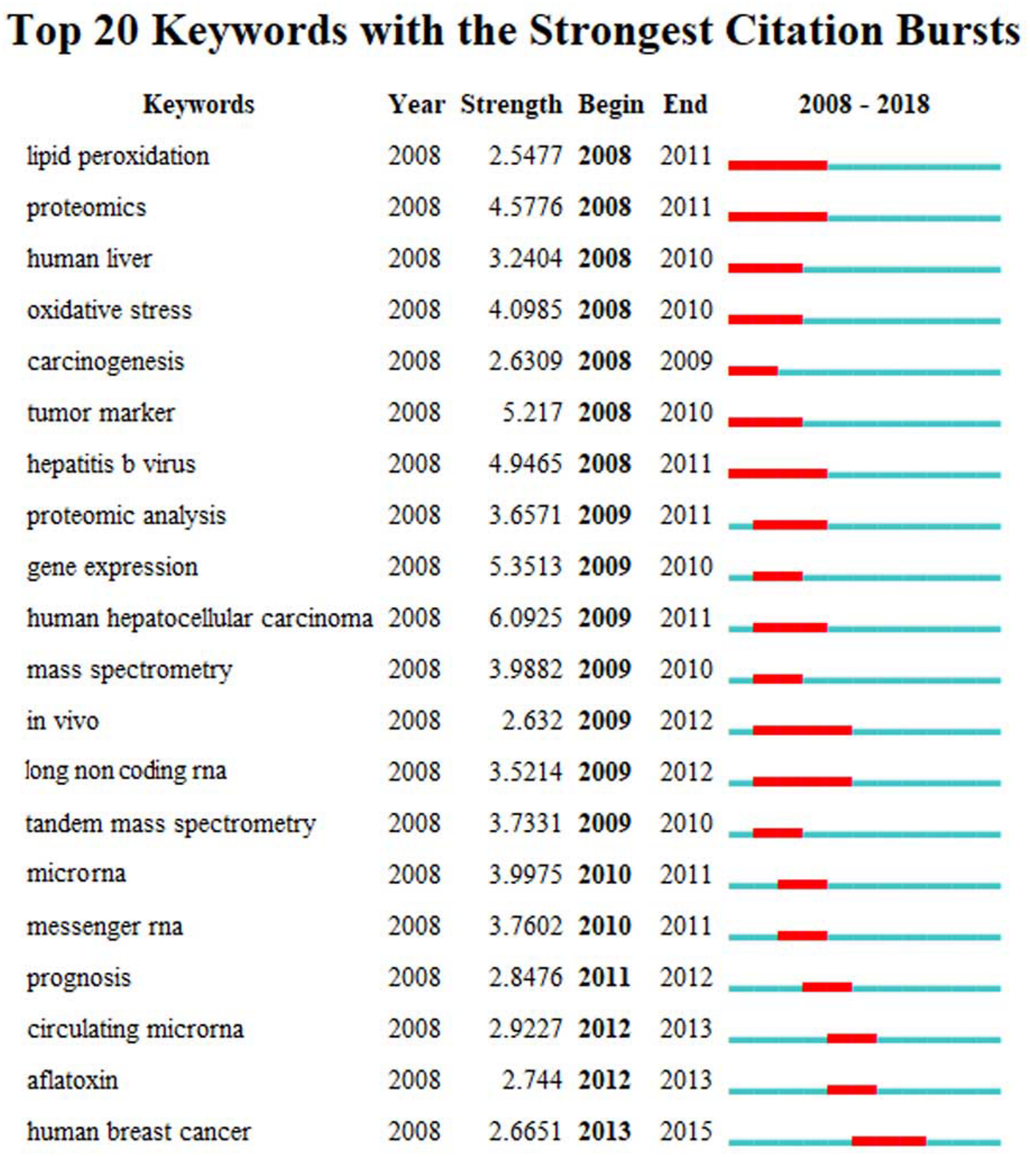
The keywords with the strongest citation bursts of publications on hepatocellular carcinoma research.

The high frequency key words were identified by CiteSpace V software analysis as the frontier research fields. One of these frontier research keywords was “lncRNA” (Fig. 3). More recently dated studies including the keyword “lncRNA” with greater frequency. Based on these results, we determined that the objectives of our study were to find the relationships between the expression levels of lncRNA and HCC progression, and to identify the potential diagnostic and prognostic biomarkers for this disease. We utilized the TCGA database HCC-related RNA sequence data mining as the data source for comparisons of gene differences and bioinformatics analysis.

### HCC-specific lncRNAs

**Figure 4:**
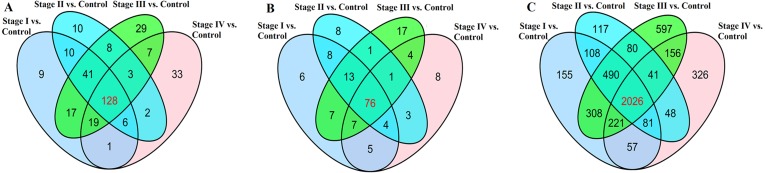
Venn diagram demonstrates the intersections of genes in TCGA data. (A) The intersection of lncRNA, (B) The intersection of miRNA, (C) The intersection of mRNA.

We found that 323 lncRNAs were significantly dysregulated in HCC tumor tissues from the TCGA database (fold change>2, *P* < 0.05). The 323 significantly dysregulated lncRNAs in HCC with different tumor stages and lymph node metastasis status were carefully analyzed. 231 lncRNAs were found to be significantly dysregulated in HCC tumor stage I/II (non-lymphatic metastasis) compared with normal liver tissues; 208 lncRNAs were significantly dysregulated in HCC tumor stage I/II (lymphatic metastasis) compared with normal liver tissues; 252 lncRNAs were significantly dysregulated in HCC tumor stage III/IV (non-lymphatic metastasis) compared with normal liver tissues; and 199 lncRNAs were significantly dysregulated in HCC tumor stage III/IV (lymphatic metastasis) compared with normal liver tissues (Fig. 4A). We selected the 128 intersected lncRNAs, which including 85 upregulated and 43 downregulated genes, for further analysis and construction of the ceRNA network (Table S1).

### Function analysis of intersected mRNAs

**Figure 5:**
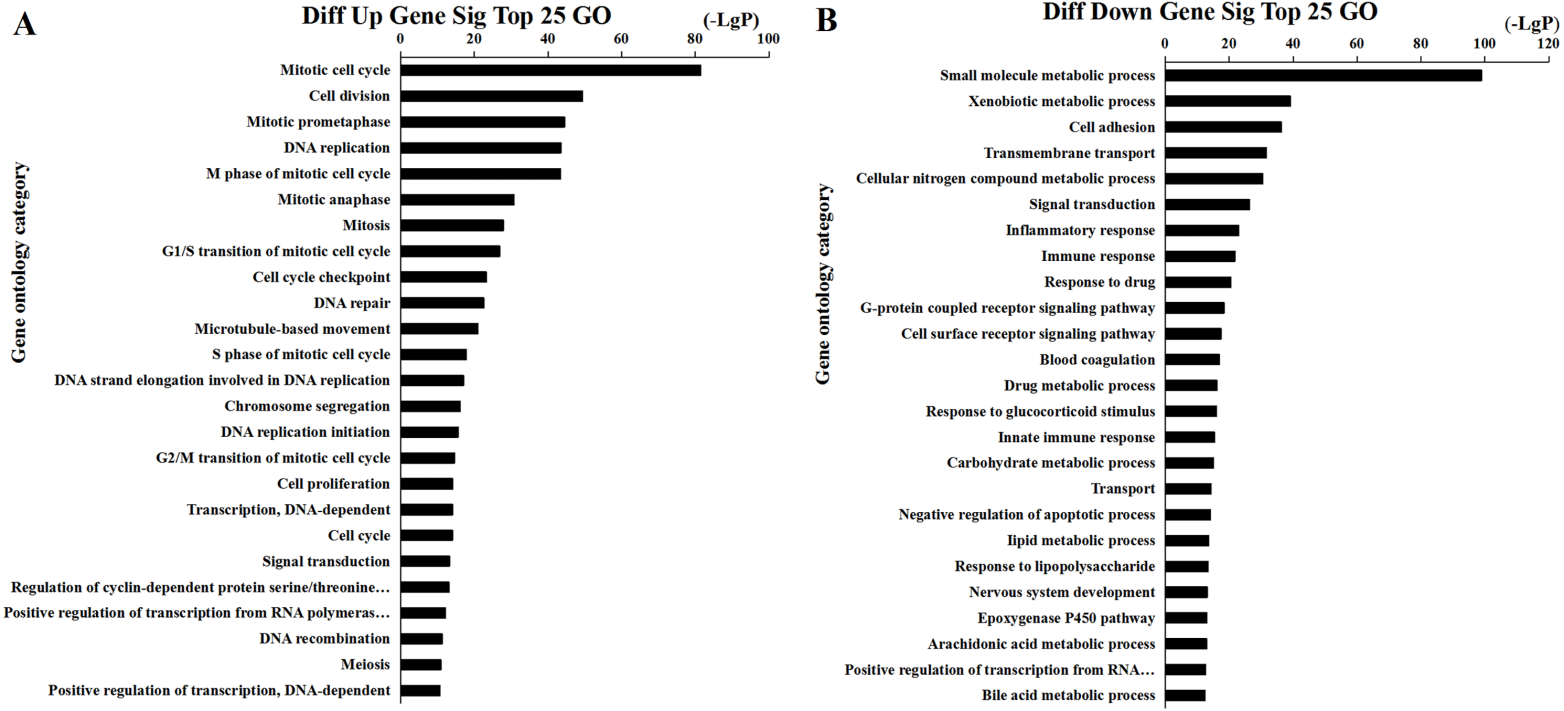
Top 25 enrichment of GO terms for HCC differentially expressed mRNAs. The bar plot shows the enrichment scores of the significant enrichment GO terms. (A) Differentially expressed up regulated genes participated significant GO (top 25), (B) Differentially expressed down regulated genes participated significant GO (top 25).

**Figure 6:**
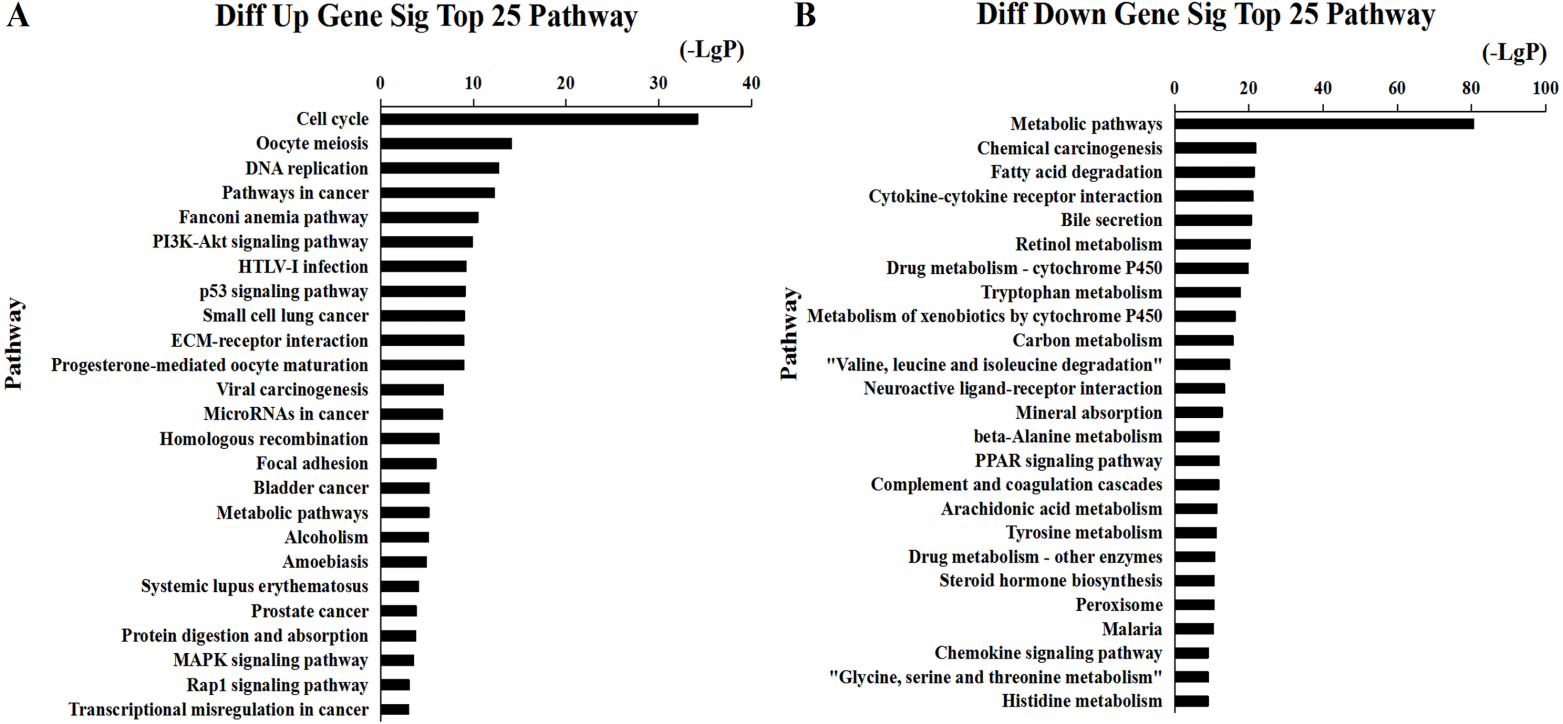
Top 25 enrichment of pathways for mRNAs HCC differentially expressed mRNAs. The bar plot shows the enrichment scores of the significant enrichment pathways. (A) Differentially expressed up regulated genes participated significant pathway (top 25), (B) differentially expressed down regulated genes participated significant pathway (top 25).

We found that 2,026 HCC tissues had significant differences in mRNA expression and were included in the Venn diagram intersection subset analysis. These differentially expressed genes may play key roles in the progression of HCC. We analyzed the potential biological regulatory functions of these 2,026 mRNA by GO enrichment of functions and KEGG pathway analyses. The most enriched function by GO analysis of upregulated mRNAs was the ‘Mitotic cell cycle’ (Fig. 5A). The most enriched function by GO analysis of downregulated mRNAs was ‘Small molecule metabolic process’ (Fig. 5B). KEGG pathway analysis indicated that 60 signaling pathways were involved in regulation by upregulated mRNAs, and the most enriched pathway was ‘Cell cycle’ (Fig. 6A). In addition, there were 152 signaling pathways involved in regulation by downregulated mRNAs, and the most enriched pathway was ‘Metabolic pathways’ (Fig. 6B). The MAPK signaling pathway has been shown to participate in the progression of HCC (Zhao et al., 2018), and the P53 signaling pathway is a key pathway in HCC cell proliferation and apoptosis (Zhao et al., 2019b). Bladder cancer, small cell lung cancer, pathways in cancer, and the PI3K-AKT signaling pathway may also be involved in the regulation of cancer progression (Tang et al., 2019).

### Prediction of miRNA targets and construction of ceRNA network

168 miRNAs were found to be significantly dysregulated between HCC tissues and normal liver tissues (fold change>2, *P* < 0.05). We selected the intersected subset of 76 miRNAs related to HCC tumor histological type and lymphatic metastasis (Fig. 4C). We predicted the potential relationships between these 76 miRNAs and the above intersected subset of 128 lncRNAs (Fig. 4A) by miRanda software. There were 59 specific miRNAs interacting with 92 specific lncRNAs (Table S2).

The ceRNA network was constructed based on the predicted miRNA-targeted genes. We predicted that miRNAs targeted mRNAs using mRBase targets and Targetscan based on the information from the 59 miRNAs described in Table S2. The intersected mRNAs were chosen from the predicted mRBase and Targetscan mRNAs. Bioinformatics was used to analyze the dysregulated intersection subset of 2026 mRNAs. 59 miRNAs were related to the 164 intersected mRNAs (Table S3).

**Figure 7:**
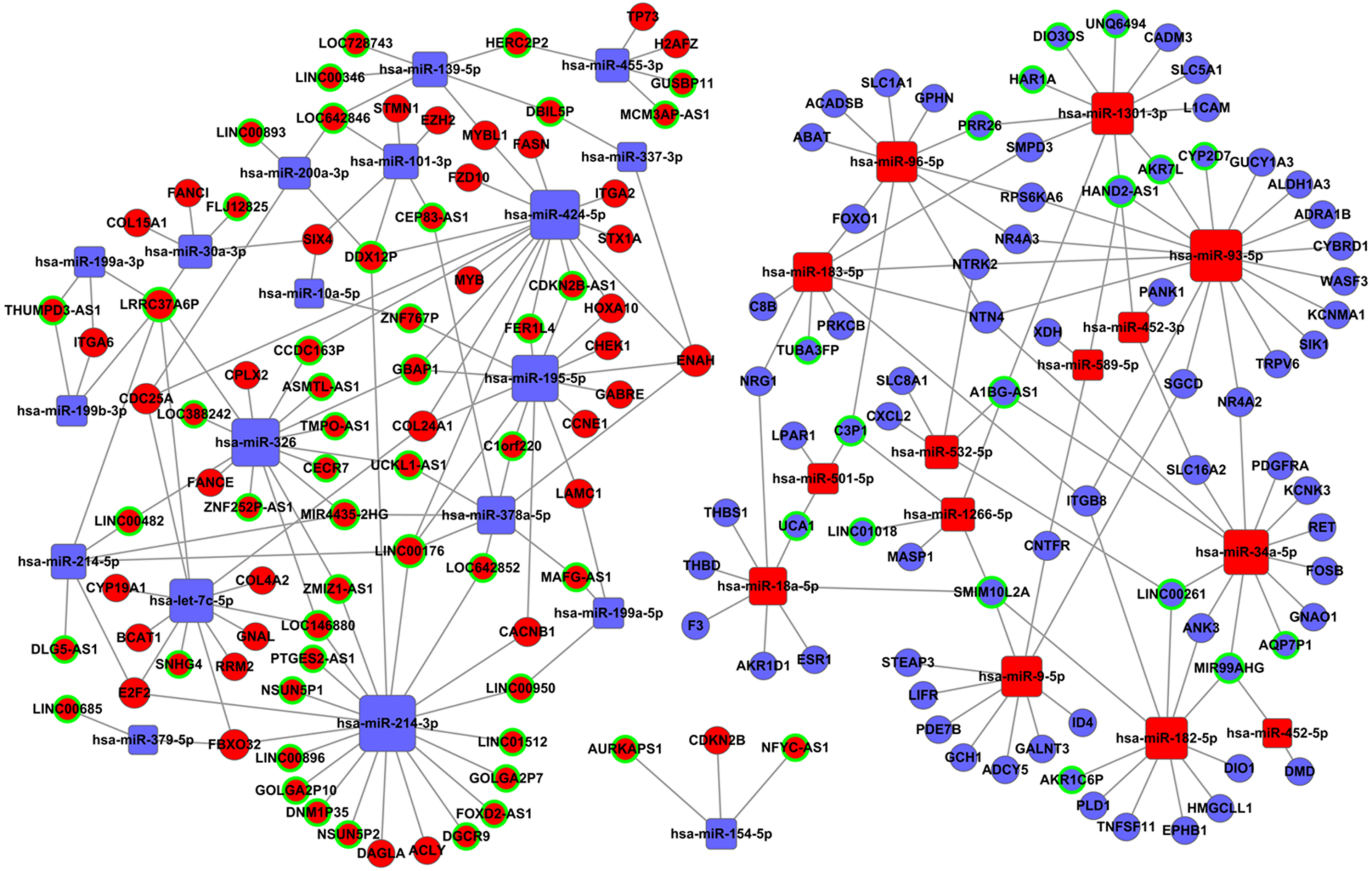
The lncRNAs-miRNAs-mRNAs ceRNA network. Red represents upregulated genes, blue represents downregulated genes; square represents miRNAs, ball represent mRNAs and ball surrounded by ring represent lncRNAs.

Tables S2 and S3 were used to construct the ceRNA network. The network was visualized using Cytoscape software 3.0. There were 66 lncRNAs, 33 miRNAs, and 93 mRNAs included in the ceRNA network (Fig. 7). The relationships among the ceRNA network genes are shown in Table S4. The DAVID database (https://david.ncifcrf.gov/mRNAs) was used to analyze genes in the ceRNA network that may be involved in the regulation of signaling pathways. The top 15 KEGG pathways, as determined by analysis of the regulatory signaling pathways, are listed in Table 2. Five cancer-related signaling pathways were enriched and categorized as pathways in cancer, small cell lung cancer, PI3K-Akt signaling pathway, p53 signaling pathway, and microRNAs in cancer. Another 10 non-cancer-related pathways were established and included metabolic pathways, ECM-receptor interaction, and aldosterone synthesis and secretion.

**Table 2:** KEGG pathways enriched by the coding mRNAs involved in the ceRNA network.

**KEGG pathways**	**Genes**
Pathways in cancer	COL4A2, LAMC1, PLD1, PRKCB, FOXO1, CCNE1, PDGFRA, RET, FZD10, ADCY5, E2F2, ITGA6, ITGA2, CDKN2B, LPAR1
Dilated cardiomyopathy	SLC8A1, CACNB1, ITGB8, SGCD, ADCY5, DMD, ITGA6, ITGA2
Arrhythmogenic right ventricular cardiomyopathy (ARVC)	SLC8A1, CACNB1, ITGB8, SGCD, ITGA6, DMD, ITGA2
Hypertrophic cardiomyopathy (HCM)	SLC8A1, CACNB1, ITGB8, SGCD, ITGA6, DMD, ITGA2
Small cell lung cancer	COL4A2, LAMC1, CCNE1, E2F2, ITGA6, ITGA2, CDKN2B
PI3K-Akt signaling pathway	COL4A2, LAMC1, CCNE1, LPAR1, THBS1, ITGB8, PDGFRA, ITGA6, ITGA2, MYB
p53 signaling pathway	STEAP3, CCNE1, TP73, THBS1, CHEK1, RRM2
Metabolic pathways	BCAT1, PANK1, CYP19A1, GALNT3, SMPD3, EPHB1, GCH1, AKR1D1, ACADSB, HMGCLL1, FASN, PLD1, E2F2, RRM2, XDH, ALDH1A3
Focal adhesion	COL4A2, LAMC1, PRKCB, THBS1, ITGB8, PDGFRA, ITGA6, ITGA2
ECM-receptor interaction	COL4A2, LAMC1, THBS1, ITGB8, ITGA6, ITGA2
GABAergic synapse	PRKCB, E2F2, GNAO1, GABRE, ADCY5, GPHN
HTLV-I infection	PDGFRA, CHEK1, FZD10, ADCY5, E2F2, CDKN2B, MYB, MYBL1
MicroRNAs in cancer	PRKCB, CDC25A, STMN1, CCNE1, THBS1, PDGFRA, EZH2, E2F2
Aldosterone synthesis and secretion	ADCY5, NR4A2, KCNK3, PRKCB, DAGLA
Gap junction	GUCY1A3, ADCY5, LPAR1, PRKCB, PDGFRA

### Associations between lncRNA signature and HCC clinical features

The 66 lncRNAs involved in the ceRNA network were selected for further investigation to identify the association between the key lncRNAs and the TCGA database of the clinicopathological features of the 313 patients with HCC. Clinicopathological features included race, gender, tumor grade, TNM stage, lymphatic metastasis, and chronic hepatitis B or C virus infection. 26 lncRNAs (13 upregulated and 13 downregulated) were found to be differentially expressed in HCC patients with different clinical features (*P* < 0.05). The results suggested that LOC642852, DDX12P, HERC2P2, UCA1, MCM3AP-AS1, CCDC163P, LINC01018, C3P1, AKR7L, AKR1C6P, A1BG-AS1, AQP7P1, UNQ6494, and LINC00261 were associated with tumor grade. CCDC163P, MCM3AP-AS1, HERC2P2, DDX12P, LOC146880, LINC01018, AKR7L, C3P1, A1BG-AS1, AKR1C6P, LINC00261, AQP7P1, and UNQ6494 were associated with TNM stages. LOC642852, CCDC163P, MAFG-AS1, THUMPD3-AS1, SMIM10L2A, AQP7P1, and PRR26 were associated with lymphatic metastasis. UCKL1-AS1, LOC146880, CCDC163P, AKR7L, and LINC01018 were associated with chronic HBV infection. UCA1, GUSBP11, C3P1, LINC00261, and GOLGA2P7 were associated with chronic HCV infection. We also found that some lncRNAs may be associated with gender or race (Table 3).

**Table 3:** The correlations between lncRNAs signature and HCC patients’ clinical characteristics in TCGA database.

**Comparisons**	**Up-regulated**	**Down-regulated**
Gender (Male vs. Female)	LOC642852, GUSBP11, HERC2P2, UCKL1-AS1, CCDC163P, FOXD2-AS1	AKR7L, CYP2D7, MIR99AHG, C3P1, AKR1C6P, LINC01018, AQP7P1, PRR26
Race (White vs. Asian)	GUSBP11, DDX12P, HERC2P2	HAND2-AS1, PRR26, C3P1, CYP2D7, AKR1C6P
Tumor grade (G III–IV vs. G I-II)	LOC642852, DDX12P, UCKL1-AS1, UCA1, MCM3AP-AS1, CCDC163P	LINC01018, C3P1, AKR7L, AKR1C6P, A1BG-AS1, AQP7P1, UNQ6494, LINC00261
TNM staging system (T3 + T4 vs. T1 + T2)	CCDC163P, MCM3AP-AS1, HERC2P2, DDX12P, LOC146880, THUMPD3-AS1	LINC01018, AKR7L, C3P1, A1BG-AS1, AKR1C6P, LINC00261, AQP7P1, UNQ6494
Lymphatic metastasis (Yes vs. No)	LOC642852, CCDC163P, MAFG-AS1, THUMPD3-AS1	SMIM10L2A, AQP7P1, PRR26
Hepatitis B virus infection	UCKL1-AS1, LOC146880, CCDC163P	AKR7L, LINC01018
Hepatitis C virus infection	UCA1, GUSBP11, GOLGA2P7	C3P1, LINC00261

### Prognostic analysis of lncRNA expression and HCC patients’ overall survival

**Figure 8:**
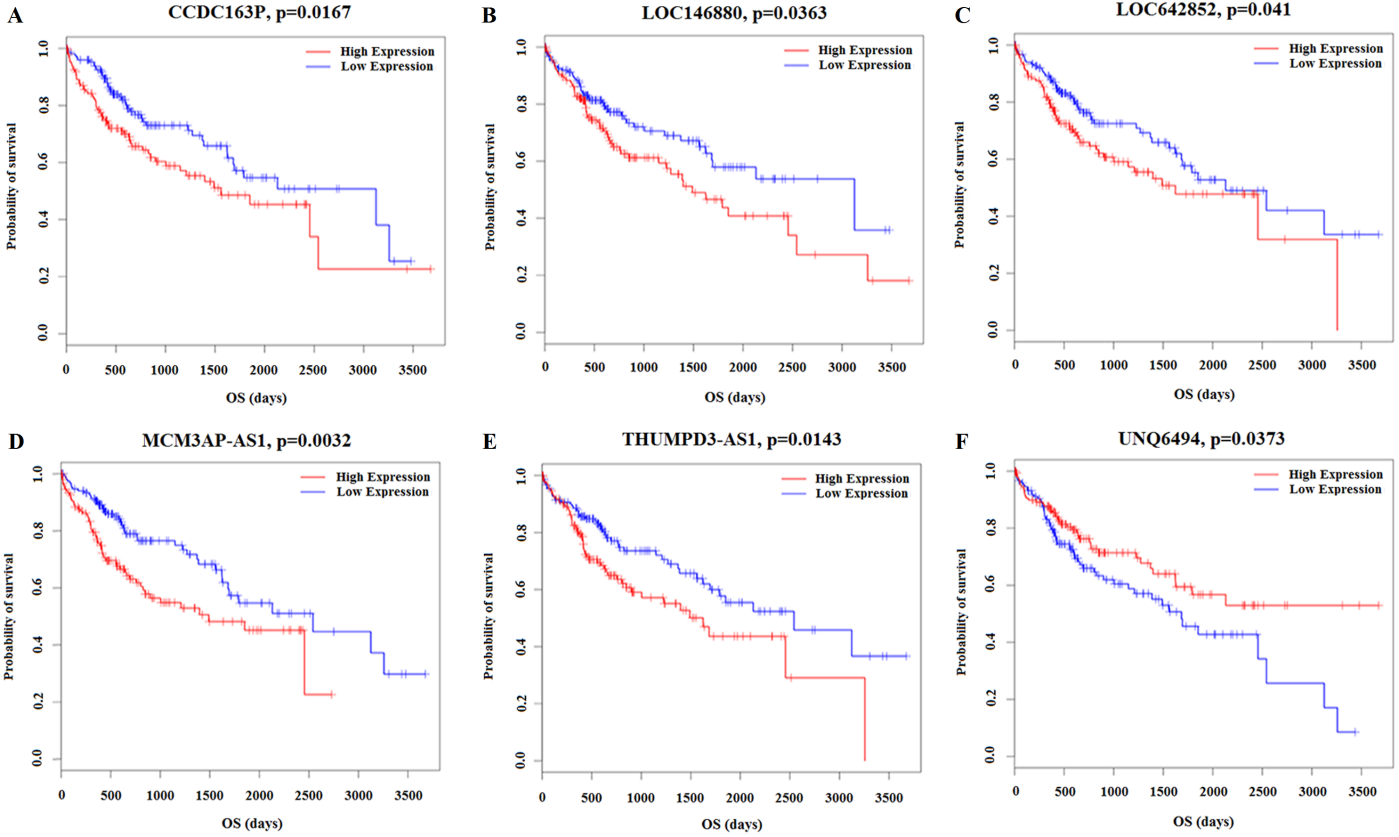
Kaplan–Meier survival curves for 6 lncRNAs associated with HCC patients overall survival time. Horizontal axis overall survival time: days, Vertical axis survival function. (A) CCDC163P; (B) LOC146880; (C) LOC642852; (D) MCM3AP-AS1; (E) THUMPD3-AS1; (F) UNQ6494.

Kaplan–Meier survival analysis was performed based on the RNA sequencing data analysis and clinical features from TCGA in HCC patients. This analysis was conducted to determine the relationships between the 26 key lncRNAs related to the clinicopathological features. The overall expression of these 26 lncRNAs in relation to HCC prognosis in TCGA was calculated using the Cox proportional hazard regression model. Six lncRNAs were associated with HCC overall survival (log-rank *P* < 0.05), and of these, CCDC163P, LOC146880, LOC642852, MCM3AP-AS1, and THUMPD3-AS1 were negatively correlated with prognosis (*P* < 0.05); UNQ6494 was positively correlated with prognosis (*P* < 0.05) (Fig. 8).

### qRT-PCR validation

**Figure 9:**
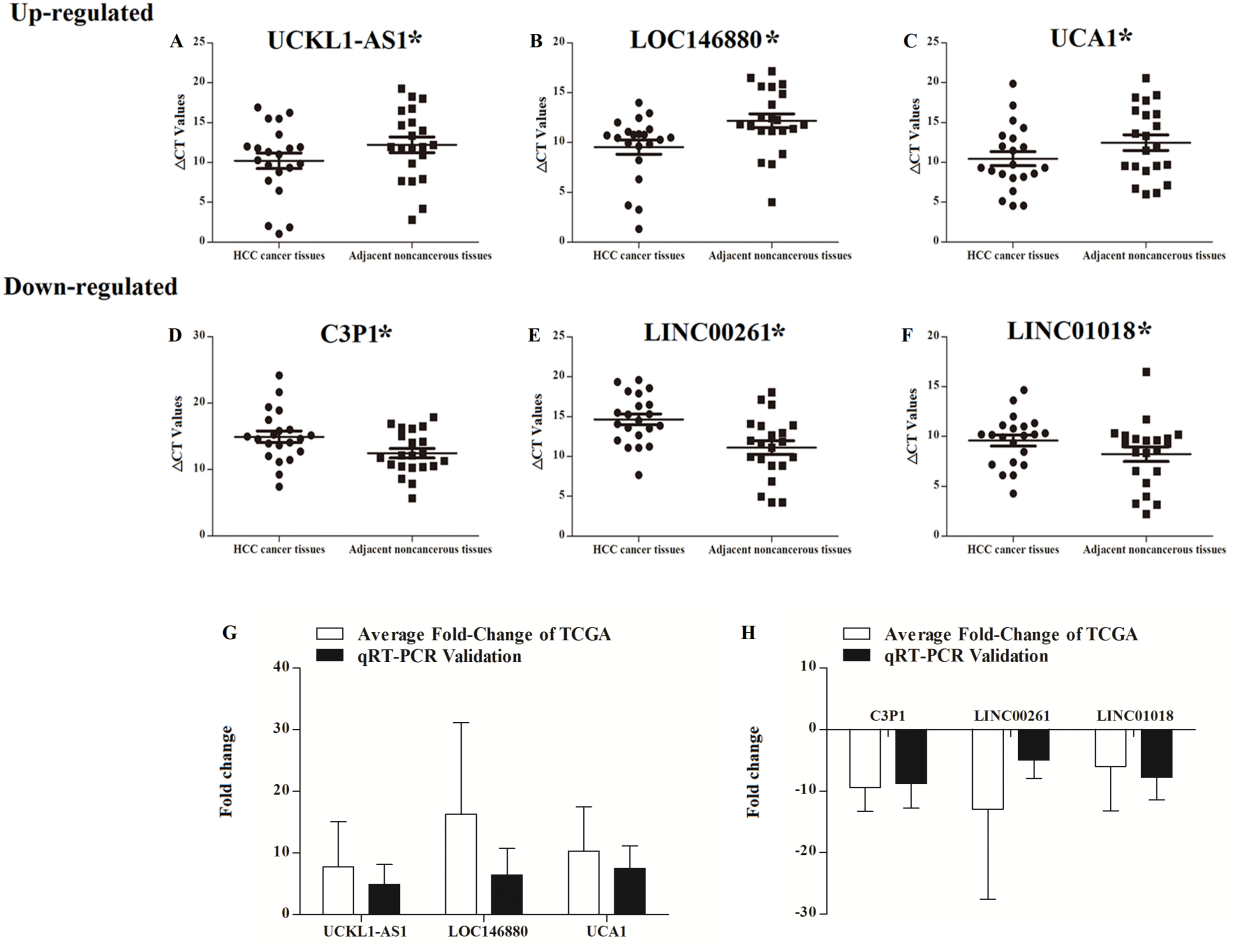
Scatter plot showing the expression levels of six lncRNAs in 20 donor HCC tissues samples. The bar plot showing the six lncRNAs HCC tissues average fold change of TCGA and qRT-PCR validation in 20 donor HCC tissues samples. A–C, up regulated lncRNAs; D–F, down regulated lncRNAs. The bar plot showing the six lncRNAs HCC tissues average fold change of TCGA and qRT-PCR validation in 20 donor HCC tissues samples (G, up regulated lncRNAs; H, down regulated lncRNAs).

UCKL1-AS1, LOC146880, UCA1, C3P1, LINC00261, and LINC01018 may be important in the progression of HCC and their expressions were detected in 20 patients with newly diagnosed HCC and their paired non-tumor liver tissue samples. qRT-PCR was used to assess the reliability and validity of our bioinformatics analysis results. UCKL1-AS1, LOC146880, and UCA1 were upregulated and C3P1, LINC00261, and LINC01018 were downregulated in HCC tissues (*P* < 0.05). qRT-PCR validation and bioinformatics analysis gave similar results in 20 newly diagnosed HCC patients (Table S1), suggesting that the bioinformatics analysis used in this study was credible (Fig. 9).

## Discussion

HCC is the predominant form of liver cancer with a high global rate of mortality (Wong et al., 2017; Zheng et al., 2018a; Zheng et al., 2018b). HCC is often diagnosed in advanced stages and has a poor prognosis (Sia et al., 2017). A growing body of evidence suggests that HBV vaccination, novel biomarkers for HCC early diagnosis, clinical therapeutic monitoring, and prognostic evaluation can control the development of HCC and improve survival (Bridges et al., 2015). The development and progression of HCC is complex and includes a variety of changes in gene expression levels and physiology. It is crucial to improve the early identification of HCC and its novel diagnostic and prognostic biomarkers. Recent advances in the understanding of lncRNAs have led to the development of potential regulatory treatments of HCC and has indicated novel biomarkers for its diagnosis and prognosis (Huo et al., 2017; Wang et al., 2019a; Wang et al., 2019b; Wang et al., 2019c). Analysis of the literature pertaining to HCC research revealed that current research is focused on lncRNA functions in tumorigenesis and tumor development. Therefore, additional large studies on tissue samples should focus on the significant differences in lncRNAs as related to HCC for use in the early diagnosis and prognosis of this disease.

The development of next-generation sequencing technology has led to the detection of thousands of dysregulated lncRNAs in various diseases (Mittempergher et al., 2019; Schmitz et al., 2016; Wang et al., 2019a; Wang et al., 2019b; Wang et al., 2019c). Recent studies have focused on the functions of differentially expressed lncRNAs and cellular regulatory mechanisms in HCC but lack sufficient clinical data to predict and evaluate the diagnostic and prognostic values of lncRNAs (Hu, Wang & Chen, 2018; Li et al., 2017; Zhou et al., 2018). Our study identified dysregulated lncRNAs in HCC by analyzing a large sample of the RNA sequencing datasets from TCGA in HCC tissues. Differentially expressed lncRNAs were identified in TCGA and ceRNA network construction was based on gene discovery. The correlation between key lncRNAs and HCC clinical features and survival was analyzed to identify potential diagnostic and prognostic biomarkers of HCC.

128 lncRNAs, 76 miRNAs, and 2026 mRNAs were found to be common differentially expressed genes in 313 HCC tumor tissues and 44 normal liver tissues according to integrated bioinformatics analysis. A portion of the 128 dysregulated lncRNAs were differentially expressed in HCC tissues or serum. Expression of downregulated lncRNA PRR26 significantly changes with the stage of HCC (Zhu et al., 2014). Lu et al. (2016a), Lu et al. (2016b) and Zhang et al. (2016) reported that lncRNA AFAP1-AS1 is upregulated in HCC tissues and promotes HCC cell proliferation and invasion and may serve as a therapeutic target. Many dysregulated lncRNAs, such as GAS5, PVT1, LINC01018, and CECR7, are differentially expressed in HCC tissues or serum and are involved in regulating disease progression (Abbastabar et al., 2018; Chang et al., 2016; Guo et al., 2018). The functions of dysregulated mRNAs were analyzed using GO and KEGG to narrow the range of genes studied. These genes expressed significant differences and intersection mRNAs with functional annotation and signal pathway regulation were used for additional bioinformatics analysis.

The ceRNAs hypothesis suggests that lncRNAs can act as ceRNAs, affecting the function of miRNA response elements and potentially regulating miRNA-related targeted gene transcription (Qian et al., 2019; Salmena et al., 2011). We constructed an HCC-related lncRNAs-miRNAs-mRNAs ceRNA network using significantly dysregulated genes from a large number of TCGA HCC tissue samples from the RNA-sequencing database. The diagnostic and prognostic biomarkers for HCC were investigated. There were 66 key lncRNAs involved in the ceRNA regulation network among the 128 HCC-related intersection lncRNAs, showing that the ceRNA network may provide key lncRNAs regulatory relationships and target genes in HCC. Many of the lncRNAs in the ceRNA network were also reported as potential diagnostic and prognostic biomarkers of HCC. For example, the HCC-related expression increase of SNHG3 can induce an epithelial-mesenchymal transition in HCC cells by miR-128, CD151 cascade axial activation, and is related to poor survival from HCC (Zhang et al., 2019). Li et al. (2019) reported that the axial regulation of LINC00346-miR-10a-5p-CDK1 may play a key role in HBV-induced HCC and LINC00346 high expression is associated with HCC poor prognosis. E2F2, BCAT1, EPHB1, RET, and LIFR, which were included in the ceRNA network, may play key roles in HCC development and progression (Farra et al., 2015; Kim et al., 2011; Luo et al., 2015; Xu et al., 2016; Ye et al., 2017). Our study analyzed 66 lncRNAs in the ceRNA network and identified 93 mRNAs indirectly involved in signaling pathways. KEGG analysis revealed that certain pathways were associated with cancer, including in small cell lung cancer, pathways in cancer, PI3K-Akt signaling pathway, p53 signaling pathway, and microRNAs in cancer (Chamcheu et al., 2019; Richardson et al., 2017; Shen et al., 2019). Our analysis revealed that the 66 key lncRNAs in the ceRNA network may play a role in the progression of HCC.

We analyzed the relationships between the expression of 66 key lncRNAs and the clinicopathological features from the TCGA database. Our results revealed 26 lncRNAs associated with these features in 313 patients with HCC. These lncRNAs were primarily associated with tumor grade, TNM stage, and lymphatic metastasis in HCC. Among these 26 lncRNAs, MCM3AP-AS1, UCA1, AKR7L, C3P1, AKR1C6P, LINC01018, and A1BG-AS1 have been reported in lymphatic metastasis and invasion and are diagnostic biomarkers for HCC (Praml, Savelyeva & Schwab, 2003; Wang et al., 2006; Zhang, Luo & Zhang, 2019; Zhao et al., 2019a; Zhao et al., 2019b; Zhao et al., 2019c; Zheng et al., 2015). However, other lncRNAs are not yet associated with HCC progression. Our study also investigated the relationships between the 26 identified lncRNAs and the overall survival in the TCGA database for HCC patients. Twelve key lncRNAs were associated with overall survival. Among these, only MCM3AP-AS1 was associated with survival in HCC (Kamel et al., 2016; Wang et al., 2019a; Wang et al., 2019b; Wang et al., 2019c; Zheng et al., 2018a; Zheng et al., 2018b). Bioinformatics analysis revealed potential novel lncRNAs biomarkers for the diagnosis, classification, and prognosis of HCC.

qRT-PCR validation of six key lncRNAs from 20 HCC tissue samples was performed to assess the accuracy and credibility of the bioinformatics results. Expression of these six lncRNAs was significantly dysregulated in 20 patients with newly diagnosed HCC and their paired non-tumor liver tissue samples. The results of the qRT-PCR validation were similar to the expression data in the TCGA database and the results of the six lncRNAs were similar to the bioinformatics analysis. Therefore, the synthetic bioinformatics analysis results are reliable.

## Conclusion

Literature metrology analysis of HCC research revealed that transcriptome- and HCC-related biomarker studies are recent research interests in HCC. We successfully identified specific HCC-associated lncRNAs from large-scale samples through the integrated analysis of RNA expression profile datasets of patients with HCC from TCGA. Differentially expressed lncRNAs and their potential functions in HCC were revealed. We investigated the specific HCC-associated lncRNAs as related to different clinicopathological features and overall survival time of patients with HCC. These ceRNA-contained key lncRNAs and are worthy of further investigation with regard to their application as biomarkers in the diagnosis, clinicopathological classification, and prognosis of patients with HCC.

##  Supplemental Information

10.7717/peerj.8758/supp-1 Table S1 Details information of HCC related differentially expressed lncRNAs in TCGA datasets 85 up-regulated, 43 down-regulated.

10.7717/peerj.8758/supp-2 Table S2 MiRNAs targeting specific intersection key lncRNAs in HCC

10.7717/peerj.8758/supp-3 Table S3 MiRNAs targeting specific intersection key mRNAs in HCC

10.7717/peerj.8758/supp-4 Table S4 Interaction between genes in ceRNA regulatory network

## Additional Information and Declarations

### Data Availability

The following information was supplied regarding data availability: Raw data are available in the Supplemental Files.
