# Supplementary material for: Integrative analysis of dysregulated lncRNA-associated ceRNA network reveals potential lncRNA biomarkers for human hepatocellular carcinoma
Source: PeerJ. 2020 Mar 11;8:e8758. doi: 10.7717/peerj.8758 (PMC7071826; doi:10.7717/peerj.8758)
Supplement: Table S1 — 85 up-regulated, 43 down-regulated. [file peerj-08-8758-s001.docx]

**Appendix: Supplementary material**

**Table S1. Details information of HCC related differentially expressed lncRNAs in TCGA datasets (85 up-regulated, 43 down-regulated)**

| **Gene ID** | **Name** | **P-value** | **FDR** | **Fold-Change** | **style** | **Gene ID** | **Name** | **P-value** | **FDR** | **Fold-Change** | **style** |
| --- | --- | --- | --- | --- | --- | --- | --- | --- | --- | --- | --- |
| 84740 | AFAP1-AS1 | 0.0000 | 0.0000 | 9.3900 | up | 79804 | HAND2-AS1 | -19.3237 | 0.0000 | 0.0128 | down |
| 284739 | LINC00176 | 0.0000 | 0.0000 | 7.9750 | up | 387742 | FAM99A | -18.5185 | 0.0000 | 0.0220 | down |
| 100048912 | CDKN2B-AS1 | 0.0000 | 0.0000 | 7.4325 | up | 255167 | LINC01018 | -14.4928 | 0.0000 | 0.0375 | down |
| 5820 | PVT1 | 0.0000 | 0.0000 | 6.9000 | up | 100132464 | FAM99B | -14.3369 | 0.0000 | 0.0218 | down |
| 92659 | MAFG-AS1 | 0.0000 | 0.0000 | 5.4550 | up | 4500 | MT1L | -10.8401 | 0.0000 | 0.0230 | down |
| 150197 | LINC00896 | 0.0000 | 0.0000 | 5.2700 | up | 285189 | PLGLA | -10.4987 | 0.0000 | 0.0375 | down |
| 286103 | ZNF252P-AS1 | 0.0000 | 0.0000 | 5.1300 | up | 100133669 | LOC100133669 | -8.5106 | 0.0000 | 0.0250 | down |
| 80307 | FER1L4 | 0.0000 | 0.0001 | 5.0550 | up | 440184 | LINC00238 | -7.0175 | 0.0000 | 0.0326 | down |
| 222699 | TOB2P1 | 0.0000 | 0.0000 | 4.7575 | up | 3580 | CXCR2P1 | -6.6667 | 0.0000 | 0.0400 | down |
| 283981 | LINC00685 | 0.0000 | 0.0000 | 4.6525 | up | 388503 | C3P1 | -6.2696 | 0.0000 | 0.0725 | down |
| 112597 | LINC00152 | 0.0001 | 0.0004 | 4.2875 | up | 652995 | UCA1 | -6.2402 | 0.0001 | 0.0427 | down |
| 100128292 | DLG5-AS1 | 0.0000 | 0.0000 | 4.2825 | up | 8755 | ADAM6 | -5.8824 | 0.0002 | 0.0557 | down |
| 100132354 | LINC01512 | 0.0000 | 0.0000 | 4.2750 | up | 256236 | NAPSB | -5.8824 | 0.0000 | 0.0525 | down |
| 8420 | SNHG3 | 0.0000 | 0.0000 | 4.1625 | up | 80350 | LPAL2 | -5.0633 | 0.0000 | 0.0900 | down |
| 155060 | LOC155060 | 0.0000 | 0.0000 | 4.0725 | up | 375719 | AQP7P1 | -4.9383 | 0.0000 | 0.0776 | down |
| 100131434 | LINC00893 | 0.0000 | 0.0000 | 3.9400 | up | 64150 | DIO3OS | -4.3478 | 0.0001 | 0.0628 | down |
| 387646 | LRRC37A6P | 0.0002 | 0.0007 | 3.8775 | up | 441432 | AQP7P3 | -4.2105 | 0.0000 | 0.0776 | down |
| 389791 | PTGES2-AS1 | 0.0000 | 0.0000 | 3.8525 | up | 342 | APOC1P1 | -3.9604 | 0.0009 | 0.0824 | down |
| 100129405 | MSTO2P | 0.0000 | 0.0000 | 3.7650 | up | 152225 | LOC152225 | -3.8095 | 0.0004 | 0.0588 | down |
| 440081 | DDX12P | 0.0000 | 0.0000 | 3.7600 | up | 114041 | B3GALT5-AS1 | -3.5714 | 0.0000 | 0.0675 | down |
| 388152 | GOLGA2P7 | 0.0000 | 0.0000 | 3.7550 | up | 503538 | A1BG-AS1 | -3.5714 | 0.0000 | 0.0800 | down |
| 100272228 | LINC00894 | 0.0000 | 0.0000 | 3.6825 | up | 414235 | PRR26 | -3.5088 | 0.0000 | 0.0851 | down |
| 541471 | MIR4435-2HG | 0.0000 | 0.0000 | 3.6150 | up | 768096 | HAR1A | -3.3898 | 0.0000 | 0.0777 | down |
| 400927 | LOC400927 | 0.0000 | 0.0000 | 3.4350 | up | 389932 | AKR1C6P | -3.3333 | 0.0013 | 0.0983 | down |
| 84793 | FOXD2-AS1 | 0.0000 | 0.0000 | 3.4250 | up | 440508 | CLEC4GP1 | -3.3333 | 0.0001 | 0.0679 | down |
| 153684 | LOC153684 | 0.0000 | 0.0000 | 3.3900 | up | 387590 | TPTEP1 | -3.3058 | 0.0000 | 0.0875 | down |
| 100130418 | CECR7 | 0.0005 | 0.0012 | 3.3575 | up | 283856 | LOC283856 | -3.2520 | 0.0000 | 0.1051 | down |
| 80161 | ASMTL-AS1 | 0.0002 | 0.0007 | 3.3200 | up | 201651 | AADACP1 | -3.1008 | 0.0007 | 0.0744 | down |
| 23642 | SNHG1 | 0.0000 | 0.0000 | 3.2975 | up | 1564 | CYP2D7 | -3.0769 | 0.0004 | 0.0852 | down |
| 338799 | LINC01089 | 0.0000 | 0.0000 | 3.2525 | up | 100129066 | UNQ6494 | -3.0303 | 0.0002 | 0.0933 | down |
| 126661 | CCDC163P | 0.0000 | 0.0000 | 3.2425 | up | 149047 | MGC27382 | -2.8986 | 0.0000 | 0.1150 | down |
| 91316 | GUSBP11 | 0.0000 | 0.0000 | 3.2375 | up | 140828 | LINC00261 | -2.8369 | 0.0000 | 0.1100 | down |
| 100128285 | DNM1P35 | 0.0000 | 0.0001 | 3.2075 | up | 84099 | ID2B | -2.7972 | 0.0000 | 0.1000 | down |
| 114043 | TSPEAR-AS2 | 0.0000 | 0.0002 | 3.1200 | up | 100188953 | LINC00092 | -2.7778 | 0.0000 | 0.0900 | down |
| 100129726 | LINC01126 | 0.0000 | 0.0000 | 3.0875 | up | 388815 | MIR99AHG | -2.6490 | 0.0001 | 0.1105 | down |
| 60674 | GAS5 | 0.0000 | 0.0000 | 3.0875 | up | 246181 | AKR7L | -2.6144 | 0.0000 | 0.1200 | down |
| 114044 | MCM3AP-AS1 | 0.0000 | 0.0000 | 3.0825 | up | 114036 | LINC00310 | -2.6144 | 0.0000 | 0.0925 | down |
| 724102 | SNHG4 | 0.0000 | 0.0000 | 3.0400 | up | 2679 | GGT3P | -2.5806 | 0.0007 | 0.1116 | down |
| 339535 | LINC01139 | 0.0017 | 0.0039 | 3.0200 | up | 113691 | TUBA3FP | -2.4691 | 0.0001 | 0.1003 | down |
| 100270804 | LOC100270804 | 0.0004 | 0.0011 | 3.0200 | up | 84848 | MIR503HG | -2.4390 | 0.0000 | 0.1052 | down |
| 260294 | NSUN5P2 | 0.0000 | 0.0000 | 2.9600 | up | 4213 | MEIS3P1 | -2.3669 | 0.0000 | 0.1175 | down |
| 6791 | AURKAPS1 | 0.0000 | 0.0000 | 2.9500 | up | 339524 | LINC01140 | -2.3392 | 0.0000 | 0.1076 | down |
| 85028 | SNHG12 | 0.0000 | 0.0000 | 2.9475 | up | 399668 | SMIM10L2A | -2.1277 | 0.0003 | 0.1033 | down |
| 100132111 | LOC100132111 | 0.0001 | 0.0002 | 2.9200 | up | 286967 | FAM223B | 0.0001 | 0.0004 | 2.5225 | up |
| 80072 | HEXA-AS1 | 0.0000 | 0.0000 | 2.9200 | up | 100131454 | DBIL5P | 0.0001 | 0.0003 | 2.5200 | up |
| 100113386 | UCKL1-AS1 | 0.0003 | 0.0010 | 2.9050 | up | 340206 | TREML3P | 0.0012 | 0.0024 | 2.5100 | up |
| 155400 | NSUN5P1 | 0.0000 | 0.0002 | 2.8550 | up | 92973 | LINC00950 | 0.0004 | 0.0012 | 2.500 | up |
| 677821 | SNORA71E | 0.0000 | 0.0002 | 2.8550 | up | 100128191 | TMPO-AS1 | 0.0000 | 0.0001 | 2.4400 | up |
| 80154 | GOLGA2P10 | 0.0000 | 0.0000 | 2.8475 | up | 100130557 | NFYC-AS1 | 0.0000 | 0.0002 | 2.4300 | up |
| 5387 | PMS2P3 | 0.0000 | 0.0002 | 2.8275 | up | 400322 | HERC2P2 | 0.0000 | 0.0001 | 2.4150 | up |
| 144486 | CEP83-AS1 | 0.0000 | 0.0000 | 2.8200 | up | 79970 | ZNF767P | 0.0000 | 0.0000 | 2.4050 | up |
| 283050 | ZMIZ1-AS1 | 0.0000 | 0.0000 | 2.8175 | up | 146880 | LOC146880 | 0.0000 | 0.0000 | 2.4025 | up |
| 400798 | C1orf220 | 0.0002 | 0.0006 | 2.7925 | up | 283487 | LINC00346 | 0.0006 | 0.0018 | 2.3900 | up |
| 349152 | DPY19L2P2 | 0.0000 | 0.0000 | 2.7875 | up | 440101 | FLJ12825 | 0.0000 | 0.0002 | 2.3825 | up |
| 25787 | DGCR9 | 0.0002 | 0.0006 | 2.7400 | up | 22973 | LAMB2P1 | 0.0003 | 0.0009 | 2.3700 | up |
| 303 | ANXA2P1 | 0.0000 | 0.0000 | 2.6800 | up | 440423 | SUZ12P1 | 0.0000 | 0.0000 | 2.3650 | up |
| 253018 | HCG27 | 0.0000 | 0.0000 | 2.6800 | up | 286467 | FIRRE | 0.0000 | 0.0000 | 2.3575 | up |
| 2630 | GBAP1 | 0.0000 | 0.0000 | 2.6725 | up | 440944 | THUMPD3-AS1 | 0.0000 | 0.0000 | 2.3300 | up |
| 653553 | HSPB1P1 | 0.0000 | 0.0000 | 2.6125 | up | 283345 | RPL13P5 | 0.0000 | 0.0001 | 2.3200 | up |
| 284185 | LINC00482 | 0.0003 | 0.0006 | 2.6075 | up | 5382 | PMS2P4 | 0.0000 | 0.0001 | 2.3075 | up |
| 388242 | LOC388242 | 0.0004 | 0.0012 | 2.5975 | up | 654434 | SNHG20 | 0.0000 | 0.0000 | 2.3000 | up |
| 642852 | LOC642852 | 0.0000 | 0.0000 | 2.5950 | up | 132241 | RPL32P3 | 0.0000 | 0.0000 | 2.2975 | up |
| 642846 | LOC642846 | 0.0000 | 0.0001 | 2.5550 | up | 304 | ANXA2P2 | 0.0000 | 0.0000 | 2.2750 | up |
| 728743 | LOC728743 | 0.0000 | 0.0000 | 2.5425 | up | 344405 | PRORSD1P | 0.0000 | 0.0000 | 2.2025 | up |
