# Supplementary material for: Integrative analysis of dysregulated lncRNA-associated ceRNA network reveals potential lncRNA biomarkers for human hepatocellular carcinoma
Source: PeerJ. 2020 Mar 11;8:e8758. doi: 10.7717/peerj.8758 (PMC7071826; doi:10.7717/peerj.8758)
Supplement: Table S2 [file peerj-08-8758-s002.docx]

**Appendix: Supplementary material**

**Table S2. MiRNAs targeting specific intersection key lncRNAs in HCC.**

| **miRNAs** | **lncRNAs** |
| --- | --- |
| hsa-let-7c-5p | HAND2-AS1, LINC00092, LOC146880, LRRC37A6P, SNHG4, SNHG4 |
| hsa-miR-101-3p | CEP83-AS1, DDX12P, LOC642846 |
| hsa-miR-10a-5p | ZNF767P |
| hsa-miR-10b-3p | LINC00950, THUMPD3-AS1 |
| hsa-miR-10b-5p | GOLGA2P10, GOLGA2P7, ZNF767P |
| hsa-miR-1180-3p | AFAP1-AS1, GOLGA2P10, GOLGA2P7, LINC00152, LINC00346, LOC146880, TOB2P1, ZNF252P-AS1 |
| hsa-miR-122-3p | LINC00346, LOC642852 |
| hsa-miR-1247-5p | HERC2P2, LINC00176 |
| hsa-miR-125b-2-3p | HAND2-AS1, LOC100270804 |
| hsa-miR-1266-5p | A1BG-AS1, C3P1, FER1L4, FOXD2-AS1, GBAP1, LAMB2P, LINC00176, LINC00346, LINC00685, LINC00950, LINC01018, LINC01512, LOC642852, LRRC37A6P, LRRC37A6P, SMIM10L2A, SNHG3, THUMPD3-AS1, TOB2P1, ZNF767P |
| hsa-miR-1269a | LRRC37A6P, MIR4435-2HG |
| hsa-miR-1301-3p | A1BG-AS1, AKR7L, ASMTL-AS1, C1orf220, DDX12P, DGCR9, DIO3OS, DNM1P35, DPY19L2P2, FOXD2-AS1, HAND2-AS1, HAR1A, HCG27, HERC2P2, LINC00346, LINC00482, LOC146880, LOC642846, LOC642852, LRRC37A6P, LRRC37A6P, NSUN5P2, PRR26, PTGES2-AS1, PVT1, UNQ6494, ZNF767P |
| hsa-miR-139-3p | ASMTL-AS1, C3P1, CEP83-AS1, DPY19L2P2, LINC00482, LINC01512, LOC155060, LOC642852, NSUN5P1, NSUN5P2, TUBA3FP |
| hsa-miR-139-5p | ADAM6, DBIL5P, HERC2P2, LINC00346, LOC642846, LOC728743 |
| hsa-miR-145-5p | C3P1, DDX12P, FLJ12825, FOXD2-AS1, GBAP1, HAR1A, HERC2P2, LINC00346, LINC00950, LINC01089, LOC155060, LOC642846, MIR99AHG, THUMPD3-AS1, ZNF767P |
| hsa-miR-154-5p | AURKAPS, NFYC-AS1 |
| hsa-miR-182-5p | AKR1C6P, GBAP1, LINC00261, MIR99AHG, SMIM10L2A |
| hsa-miR-183-5p | FER1L4, HERC2P2, LINC00346, LOC153684, MSTO2P, THUMPD3-AS1, TUBA3FP |
| hsa-miR-18a-5p | CCDC163P, FIRRE, LINC00894, LINC00950, MAFG-AS1, SMIM10L2A, UCA1 |
| hsa-miR-195-5p | C1orf220, CDKN2B-AS1, CDKN2B-AS1, FER1L4, GBAP1, LINC00176, NAPSB, SMIM10L2A, ZNF767P |
| hsa-miR-199a-3p | LRRC37A6P, MIR99AHG, THUMPD3-AS1 |
| hsa-miR-199a-5p | LINC00950, MAFG-AS1, PRR26 |
| hsa-miR-199b-3p | LRRC37A6P, MIR99AHG, THUMPD3-AS1 |
| hsa-miR-19a-3p | LINC00950 |
| hsa-miR-200a-3p | DDX12P, DDX12P, LINC00893, LOC642846 |
| hsa-miR-214-3p | A1BG-AS1, CXCR2P1, DDX12P, DGCR9, DNM1P35, FOXD2-AS1, GGT3P, GOLGA2P10, GOLGA2P7, HAR1A, LINC00176, LINC00310, LINC00896, LINC00950, LINC01512, LOC146880, LOC642852, MEIS3P1, MT1L, NAPSB, NSUN5P1, NSUN5P2, PTGES2-AS1, SMIM10L2A, TPTEP1, ZMIZ1-AS1 |
| hsa-miR-214-5p | C3P1, DLG5-AS1, LINC00176, LINC00482, LRRC37A6P, MIR4435-2HG |
| hsa-miR-221-3p | LOC146880 |
| hsa-miR-224-5p | ANXA2P1, CDKN2B-AS1 |
| hsa-miR-30a-3p | FLJ12825, LRRC37A6P |
| hsa-miR-30c-2-3p | C3P1, DNM1P35, FER1L4, HAND2-AS1, LINC00950, LINC01140, LINC01512, LOC153684, LOC728743, MIR4435-2HG, MIR503HG, MIR99AHG, NSUN5P1, NSUN5P2, NF767P |
| hsa-miR-326 | ASMTL-AS1, CCDC163P, CECR7, GBAP1, HAND2-AS1, HAR1A, LINC00261, LINC00482, LOC146880, LOC388242, LRRC37A6P, MIR4435-2HG, MIM10L2A, TMPO-AS1, UCKL1-AS1, ZMIZ1-AS1, ZNF252P-AS1 |
| hsa-miR-337-3p | ADAM6, DBIL5P |
| hsa-miR-34a-5p | A1BG-AS1, A1BG-AS1, AQP7P1, CCDC163P, LINC00176, LINC00261, LINC00346, MIR99AHG, TOB2P1, UCKL1-AS1 |
| hsa-miR-3653-3p | ZNF252P-AS1 |
| hsa-miR-375 | DBIL5P, HERC2P2 |
| hsa-miR-378a-5p | C1orf220, CEP83-AS1, LINC00176, LINC00261, LOC642852, MAFG-AS1, MEIS3P1, MIR4435-2HG, SMIM10L2A, UCKL1-AS1 |
| hsa-miR-379-5p | LINC00685 |
| hsa-miR-383-5p | CDKN2B-AS1, LINC00893, LOC728743, MCM3AP-AS1 |
| hsa-miR-411-5p | ANXA2P2 |
| hsa-miR-424-3p | LOC642852 |
| hsa-miR-424-5p | CCDC163P, CDKN2B-AS1, DDX12P, FER1L4, GBAP1, LINC00176 |
| hsa-miR-452-3p | ASMTL-AS1, GUSBP11, HAND2-AS1, LOC642852, LRRC37A6P, MCM3AP-AS1 |
| hsa-miR-452-5p | HERC2P2, MIR99AHG |
| hsa-miR-455-3p | A1BG-AS1, GUSBP11, HERC2P2, MCM3AP-AS1 |
| hsa-miR-483-3p | AQP7P1, FLJ12825, GOLGA2P10, GOLGA2P7, LINC00346, LOC155060, LRRC37A6P, SMIM10L2A |
| hsa-miR-483-5p | ZNF252P-AS1 |
| hsa-miR-486-5p | LINC00176, LINC00482 |
| hsa-miR-490-3p | AKR1C6P, CCDC163P, DNM1P35, FER1L4, GUSBP11, HAR1A, LINC00950, LOC642852, SNHG20 |
| hsa-miR-500a-3p | AFAP1-AS1, FER1L4, HERC2P2, LOC728743, ZNF252P-AS1 |
| hsa-miR-501-5p | C3P1, GOLGA2P10, GOLGA2P7, HERC2P2, LINC00894, UCA1, UCKL1-AS1 |
| hsa-miR-532-5p | A1BG-AS1, LINC00261 |
| hsa-miR-542-5p | FER1L4, GUSBP11 |
| hsa-miR-589-5p | AFAP1-AS1, FER1L4, HAND2-AS1, LOC642852, LOC728743 |
| hsa-miR-654-3p | C1orf220, CDKN2B-AS1, FER1L4, FLJ12825, MCM3AP-AS1 |
| hsa-miR-758-3p | DNM1P35 |
| hsa-miR-93-5p | AKR7L, C1orf220, CYP2D7, DLG5-AS1, FIRRE, HAND2-AS1, HEXA-AS1, PVT1, SNHG20 |
| hsa-miR-9-5p | LINC00893, SMIM10L2A, TOB2P1, ZNF252P-AS1 |
| hsa-miR-96-5p | C3P1, FER1L4, HERC2P2, LOC153684, LRRC37A6P, PRR26 |
