# Supplementary material for: Integrative analysis of dysregulated lncRNA-associated ceRNA network reveals potential lncRNA biomarkers for human hepatocellular carcinoma
Source: PeerJ. 2020 Mar 11;8:e8758. doi: 10.7717/peerj.8758 (PMC7071826; doi:10.7717/peerj.8758)
Supplement: Table S3 [file peerj-08-8758-s003.docx]

**Appendix: Supplementary material**

**Table S3. MiRNAs targeting specific intersection key mRNAs in HCC**

| **miRNAs** | **mRNAs** |
| --- | --- |
| hsa-let-7c-5p | BCAT1, CCL3, CDC25A, COL24A1, COL4A2, CYP19A1, DMD, E2F2, FBXO32, GDF6, GNAL, IGF1, ITGB8, MASP1, RRM2, STEAP3, THBS1, TMPRSS2 |
| hsa-miR-101-3p | ACVR1C, ANK3, EZH2, FOS, ITGB8, JUNB, LIFR, NR4A3, SIX4, STMN1 |
| hsa-miR-10a-5p | ALPL, NR4A3, SIX4 |
| hsa-miR-10b-3p | BCAT1, EHHADH, SEMA6D |
| hsa-miR-10b-5p | ALPL, NR4A3 |
| hsa-miR-1258 | E2F1 |
| hsa-miR-125b-2-3p | EHHADH, GPHN, NEGR1, PTGS2 |
| hsa-miR-1266-5p | E2F2, MASP1 |
| hsa-miR-1269a | FOXO1, GABRE |
| hsa-miR-1301-3p | CADM3, EZH2, L1CAM, SIX4, SLC5A1, SMPD3 |
| hsa-miR-130a-3p | CHRM2, ESR1, ITGB8, MYBL1, PDGFRA, SIK1, TGFA |
| hsa-miR-139-5p | DMD, FOS, FOXO1, GALNT3, MYBL1, SOCS2 |
| hsa-miR-142-5p | SLC22A3 |
| hsa-miR-144-3p | FOSB, MYBL1, SLC45A3, TNFSF11 |
| hsa-miR-145-5p | ADRA2B, ITGB8, NTN4, RPS6KA6, SLC7A8, TLR4 |
| hsa-miR-154-5p | ANK3, CDKN2B |
| hsa-miR-182-5p | ANK3, CACNB4, DIO1EPHB1, GABRE, HMGCLL1, ITGB8, PLD1, TNFSF11 |
| hsa-miR-183-5p | C8B, ENAH, FOXO1, ITGB8, NRG1, NTN4, NTRK2, PRKCB, SMPD3 |
| hsa-miR-18a-5p | AKR1D1, ESR1, F3, NRG1, THBD, THBS1 |
| hsa-miR-195-5p | CACNB1, CCNE1, CHEK1, COL24A1, ENAH, FOXO1, GABRE, GHR, HOXA10, LAMC1, MASP1, PAK7, RPS6KA6, SEMA3D, SEMA6D, SIK1 |
| hsa-miR-199a-3p | DUSP5, ITGA6, ITGB8, PRKCB, ST6GAL2 |
| hsa-miR-199a-5p | LAMC1, PPARGC1A, WNT2 |
| hsa-miR-199b-3p | DUSP5, ITGA6, ITGB8, PRKCB, ST6GAL2 |
| hsa-miR-19a-3p | BCAT1, DBT, NTRK2, SGCD |
| hsa-miR-200a-3p | CDC25A, SIK1 |
| hsa-miR-21-5p | CNTFR, ENAH, TNFRSF11B |
| hsa-miR-214-3p | ACLY, CACNB1, CTF1, DAGLA, E2F2, FBXO32, KCNK3, NTRK2, SEMA3D, SGCD |
| hsa-miR-214-5p | E2F2 |
| hsa-miR-221-3p | CACNB4, CD4, CXCL12, FOS, ITGB8, MYBL1, NCAM1, SOCS3 |
| hsa-miR-222-3p | FOS, NRG1SCD5, THBS1 |
| hsa-miR-224-3p | CNTFR, IL1RAP |
| hsa-miR-224-5p | ACVR1C, ANK3, ENAH, FOSB, GABRE, KCNMA1, NR4A3 |
| hsa-miR-30a-3p | COL15A1, FANCI, KYNU, NTRK3, SEMA3E, SIX4, TGFA |
| hsa-miR-30c-2-3p | ATP1B2, CADM3, CXCL12, NTRK3, TMPRSS13 |
| hsa-miR-326 | CPLX2, FANCE, KHK, NGFR, UROC1 |
| hsa-miR-335-5p | ACVR1C, PGR, SLC45A3 |
| hsa-miR-337-3p | ENAH |
| hsa-miR-33b-5p | ANK3, CAMK4, GHR, PDGFRA, SIK1 |
| hsa-miR-34a-5p | ANK3, CACNB1, CCNE2, CPLX2, FOSB, GNAO1, KCNK3, MAPT, NR4A2, NTN4, PDGFRA, RET, SLC16A2, STX1A, TP73 |
| hsa-miR-3607-3p | CDC7, GALNT3, NRG1, TNXB |
| hsa-miR-375 | SLC16A2 |
| hsa-miR-378a-5p | ACADS, ENAH, IGF2, PGR, RPS6KA6 |
| hsa-miR-379-5p | FBXO32, MASP1 |
| hsa-miR-424-5p | CDC25A, COL24A1, ENAH, FASN, FZD10, GHR, GLS2, HOXA10, ITGA2, MASP1, MYB, MYBL1, ANK1, RPS6KA6, SLC4A4, STX1A |
| hsa-miR-452-3p | PANK1, SLC16A2 |
| hsa-miR-452-5p | DMD |
| hsa-miR-455-3p | ADCY1, H2AFZ, TGFA, TP73 |
| hsa-miR-486-5p | EPHA3, FOXO1, GABRB3 |
| hsa-miR-490-3p | TGFA |
| hsa-miR-500a-3p | CAMK4, CDKN2A, SOCS2 |
| hsa-miR-501-3p | HOXD10 |
| hsa-miR-501-5p | LPAR1 |
| hsa-miR-532-5p | CACNB4, CXCL2, NTRK2, SLC8A1 |
| hsa-miR-589-5p | CNTFR, XDH |
| hsa-miR-758-3p | PDE7B |
| hsa-miR-9-5p | ADCY5, CCNE2, CNTFR, COL15A1, COL9A1, GAD1, GALNT3, GCH1, ID4, LIFR, LMNA, PDE7B, SGCD, STEAP3 |
| hsa-miR-93-5p | ADRA1B, ALDH1A3, ARHGEF11, CYBRD1, DAPK2, E2F1, E2F2, FGD1, GUCY1A3, ITGB8, CNMA1, KIF23, NR4A2, NR4A3, NTN4, NTRK2, PLA2G6, RPS6KA6, SGCD, SIK1, TRPV6, WASF3 |
| hsa-miR-96-5p | ABAT, ACADSB, B4GALNT1, CACNB4, FBXO32, FOXO1, GPHN, NR4A3, NTN4, RPS6KA6, SLC1A1 |
