# Supplementary material for: Integrative analysis of dysregulated lncRNA-associated ceRNA network reveals potential lncRNA biomarkers for human hepatocellular carcinoma
Source: PeerJ. 2020 Mar 11;8:e8758. doi: 10.7717/peerj.8758 (PMC7071826; doi:10.7717/peerj.8758)
Supplement: Table S4 [file peerj-08-8758-s004.docx]

**Appendix: Supplementary material**

**Table S4. Interaction between genes in ceRNA regulatory network**

| **mRNAs** | **miRNAs** | **lncRNAs** |
| --- | --- | --- |
| BCAT1, CDC25A, COL24A1, COL4A2, CYP19A1, E2F2, FBXO32, GNAL, RRM2 | hsa-let-7c-5p | LOC146880, LRRC37A6P, SNHG4 |
| EZH2, SIX4, STMN1 | hsa-miR-101-3p | CEP83-AS1, DDX12P, LOC642846 |
| SIX4 | hsa-miR-10a-5p | ZNF767P |
| MASP1 | hsa-miR-1266-5p | A1BG-AS1, C3P1, LINC01018, SMIM10L2A |
| CADM3, L1CAM, SLC5A1, SMPD3 | hsa-miR-1301-3p | A1BG-AS1, AKR7L, DIO3OS, HAND2-AS1, HAR1A, PRR26, UNQ6494 |
| MYBL1 | hsa-miR-139-5p | DBIL5P, HERC2P2, LINC00346, LOC642846, LOC728743 |
| CDKN2B | hsa-miR-154-5p | AURKAPS1, NFYC-AS1 |
| ANK3, DIO1, EPHB1, HMGCLL1, ITGB8, PLD1, TNFSF11 | hsa-miR-182-5p | AKR1C6P, LINC00261, MIR99AHG, SMIM10L2A |
| C8B, FOXO1, ITGB8, NRG1, NTN4, NTRK2, PRKCB, SMPD3 | hsa-miR-183-5p | TUBA3FP |
| AKR1D1, ESR1, F3, NRG1, THBD, THBS1 | hsa-miR-18a-5p | SMIM10L2A, UCA1 |
| CACNB1, CCNE1, CHEK1, COL24A1, ENAH, GABRE, HOXA10, LAMC1 | hsa-miR-195-5p | C1orf220, CDKN2B-AS1, FER1L4, GBAP1,  LINC00176, ZNF767P |
| ITGA6 | hsa-miR-199a-3p | LRRC37A6P, THUMPD3-AS1 |
| LAMC1 | hsa-miR-199a-5p | LINC00950, MAFG-AS1 |
| ITGA6 | hsa-miR-199b-3p | LRRC37A6P, THUMPD3-AS1 |
| CDC25A | hsa-miR-200a-3p | DDX12P, LINC00893, LOC642846 |
| ACLY, CACNB1, DAGLA, E2F2, FBXO32 | hsa-miR-214-3p | DDX12P, DGCR9, DNM1P35, FOXD2-AS1, GOLGA2P10, GOLGA2P7, LINC00176, LINC00896, LINC00950, LINC01512, LOC146880, LOC642852, NSUN5P1, NSUN5P2, PTGES2-AS1, ZMIZ1-AS1 |
| E2F2 | hsa-miR-214-5p | DLG5-AS1, LINC00176, LINC00482, LRRC37A6P, MIR4435-2HG |
| COL15A1, FANCI, SIX4 | hsa-miR-30a-3p | FLJ12825, LRRC37A6P, |
| CPLX2, FANCE | hsa-miR-326 | ASMTL-AS1, CCDC163P, CECR7, GBAP1, LINC00482,  LOC146880, LOC388242, LRRC37A6P, MIR4435-2HG, TMPO-AS1, UCKL1-AS1, ZMIZ1-AS1, ZNF252P-AS1 |
| ENAH | hsa-miR-337-3p | DBIL5P |
| ANK3, FOSB, GNAO1, KCNK3, NR4A2, NTN4, PDGFRA, RET, SLC16A2 | hsa-miR-34a-5p | A1BG-AS1, AQP7P1, LINC00261, MIR99AHG |
| ENAH | hsa-miR-378a-5p | C1orf220, CEP83-AS1, LINC00176, LOC642852, MAFG-AS1, MIR4435-2HG, UCKL1-AS1 |
| FBXO32 | hsa-miR-379-5p | LINC00685 |
| CDC25A, COL24A1, ENAH, FASN, FZD10, HOXA10, ITGA2, MYB, MYBL1, STX1A | hsa-miR-424-5p | CCDC163P, CDKN2B-AS1, DDX12P, FER1L4, GBAP1, LINC00176 |
| PANK1, SLC16A2 | hsa-miR-452-3p | HAND2-AS1 |
| DMD | hsa-miR-452-5p | MIR99AHG |
| H2AFZ, TP73 | hsa-miR-455-3p | GUSBP11, HERC2P2, MCM3AP-AS1 |
| LPAR1 | hsa-miR-501-5p | C3P1, UCA1 |
| CXCL2, NTRK2, SLC8A1 | hsa-miR-532-5p | A1BG-AS1, LINC00261 |
| CNTFR, XDH | hsa-miR-589-5p | HAND2-AS1 |
| ADCY5, CNTFR, GALNT3, GCH1, ID4, LIFR, PDE7B, SGCD, STEAP3 | hsa-miR-9-5p | SMIM10L2A |
| ADRA1B, ALDH1A3, CYBRD1, GUCY1A3, ITGB8, KCNMA1, NR4A2, NR4A3, NTN4, NTRK2, RPS6KA6, SGCD, SIK1, TRPV6, WASF3 | hsa-miR-93-5p | AKR7L, CYP2D7, HAND2-AS1 |
| ABAT, ACADSB, FOXO1, GPHN, NR4A3, NTN4, RPS6KA6, SLC1A1 | hsa-miR-96-5p | C3P1, PRR26 |
